# Intrathoracic Migration of a Breast Prosthesis after Minimally-Invasive Atrial Myxoma Resection: A Case Report and Literature Review

**DOI:** 10.1055/a-2819-1883

**Published:** 2026-05-29

**Authors:** Amy Dawson, Emily Mackay, Gordon Buduhan

**Affiliations:** 1 Department of General Surgery, Memorial University, St. John's, Newfoundland and Labrador, Canada; 2 Division of Thoracic Surgery, University of British Columbia, Vancouver, British Columbia, Canada; 3 Division of Thoracic Surgery, Department of Surgery, University of British Columbia, Kelowna, British Columbia, Canada

**Keywords:** breast prosthesis, breast implant, minimally invasive cardiothoracic surgery, atrial myxoma, intrathoracic migration

## Abstract

Intrathoracic migration of breast prostheses has been described after both open and minimally invasive cardiothoracic and breast surgery, with several proposed mechanisms of migration. In this report, we describe an unusual presentation of intrathoracic migration of a breast prosthesis following remote minimally invasive cardiac surgery for atrial myxoma resection. Due to the rare but documented risk of intrathoracic breast prosthesis migration following minimally invasive cardiothoracic surgery, consideration should be given to strategies for mitigating this risk at the time of surgery.

## Introduction

Intrathoracic breast prosthesis migration has been previously described following surgical interventions where the chest wall was deliberately or unintentionally compromised ( Table 1 ). The first reported case followed suspicion of intraoperative chest wall compromise during breast augmentation. 1 Other cases after breast augmentation have been described, where migration of the prosthesis through an inadvertent chest wall defect occurred after external pressure, such as postoperative breast massage. 2 3 Other cases have been described after minimally invasive mitral valve surgery. 4 5 More commonly, intrathoracic migration of breast prostheses has been described after surgeries where the thoracic cavity has been deliberately accessed through the chest wall, usually for thoracoscopic or open cancer resection. 6 7 8 9 10 11 12 13 It is hypothesized that the normal negative intrapleural pressure, alone or augmented with chest tube placement, is sufficient to draw a mobile breast prosthesis through the chest wall defect. This case report describes the first reported presentation of an intrathoracic migration of a breast prosthesis following minimally invasive atrial myxoma surgery. Notably, there have been only two prior case reports of intrathoracic breast prosthesis migration after minimally invasive cardiac surgery. This case, therefore, significantly contributes to the limited body of literature on this rare complication of breast prosthesis implantation.

**Table 1:** Summary of relevant literature

Authors	Date	Location	Patient age (years)	Diagnosis	Type of prosthesis	Surgical intervention	Post/Intraoperative complications	Precipitating factors to migration	Time to prosthesis migration
Chen et al 1	2005	Qingdao University Medical School, Qingdao, China	29	None	Silicone breast implants	Bilateral breast augmentation, transaxillary approach	Sudden dyspnea during left-sided operation, resolved with administration of oxygen	Left breast massage	2 months
Mehta et al 8	2008	Kennemer Hospital, Haarlem, The Netherlands	52	Right upper lobe T1N0M0 non-small cell lung cancer	Silicone breast implant	Muscle-sparing thoracotomy, right upper lobectomy	Chyle leak requiring prolonged chest tube drainage	Hypothesized to be negative intrathoracic pressure secondary to the chest tube	12 days
Kim et al 3	2009	Seoul National University Bundang Hospital, Korea	34	None	Not specified	Right Breast augmentation revision by transaxillary approach	Workup for pneumothorax postoperatively, no complications found	Breast massage at the local clinic	1 month
Fong and Hoffmann 5	2011	Johns Hopkins University, Baltimore, MD, United States	59	Severe mitral regurgitation	Not specified	Minimally invasive mitral valve repair	None reported	Pilates stretching exercise	“Recent,” not specified
Lee et al 2	2011	Chung Ang University Hospital, Seoul, South Korea	23	None	Cohesive silicone gel breast implant	Augmentation mammoplasty	Mild intermittent chest pain	Breast massage	6 days
Sykes and Rosella 9 a	2012	University of Rochester Medical Center, Rochester, NY, United States	72	Right middle lobe grade I, well-differentiated carcinoid tumor	Silicone breast implant	Video-associated thoracoscopic right middle lobectomy	None reported	None reported	6 months
Lehoux et al 10 a	2013	University of Rochester Medical Center, Rochester, NY, United States	71	Right middle lobe carcinoid tumor	Silicone breast implant	Video-associated thoracoscopic right middle lobectomy	None reported	None reported	6 months
Bruintjes et al 7	2014	Canisius Wilhelmina Hospital, Nijmegen, The Netherlands	51	COPD, non-specific necrotizing inflammation	Silicone breast implant	Video-associated thoracoscopic right upper lobe wedge resection	None reported	Hypothesized to be physiologic negative intrathoracic pressure	Postoperative period, not specified
Roussel et al 11 a	2015	University of Rochester Medical Center, Rochester, NY, United States	72	Right middle lobe carcinoid tumor	Silicone breast implant	Video-associated thoracoscopic right middle lobectomy	None reported	Pilates stretching maneuver	6 months
Songcharoen et al 4	2015	University of Mississippi Medical Centre, Jackson, MS, United States	61	Not specified	Inflatable saline implants	Minimally invasive mitral valvuloplasty	None reported	None reported	5 months
Febbo et al 12	2021	Rush University Medical Centre, Chicago, IL, United States	60	Stage I right upper lobe lung adenocarcinoma	Silicone breast implant	Video-associated thoracoscopic right upper lobectomy	Pneumothorax	None reported	≤6 months
Damirov et al 6	2022	University of Munich, Munich, Germany	52	Right upper lobe lung adenocarcinoma	Not specified	Thoracotomy, right upper lobectomy	None reported	None reported	7 years
Stewart and Thomas 13	2024	University of Kansas School of Medicine, Kansas City, KS, United States	73	Breast cancer; non-small cell lung cancer	Silicone breast implant	Double mastectomy with reconstruction; superior segmentectomy of the right lower lung via open thoracotomy	None reported	Pulmonary function testing	3 years (post-open thoracotomy)

a These cases are reported from the same center with similar presentations, and are likely to represent the same patient.

## Case

**Fig. 1:**
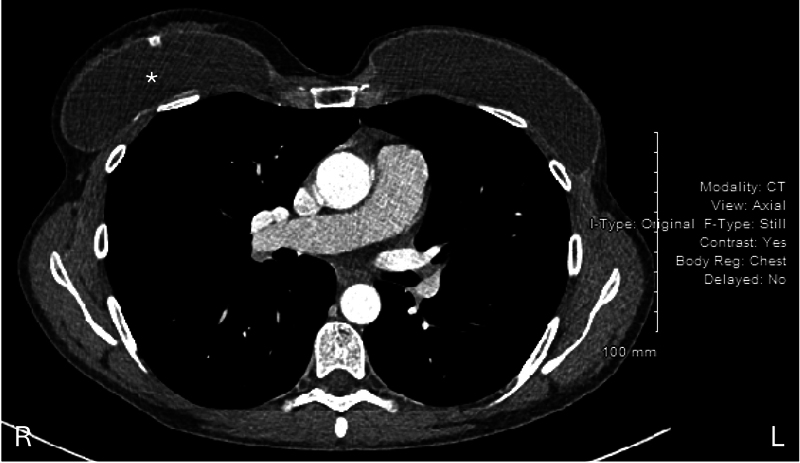
CT imaging demonstrating the right prosthesis (*) in the original position.

A 50-year-old female with a history of breast carcinoma presented with an intact breast prosthesis in the right posterior basal pleural space. She was initially diagnosed in 2007 with an invasive ductal left breast carcinoma treated with mastectomy and adjuvant chemoradiation. She underwent left breast reconstruction and right breast augmentation with bilateral saline breast implants (Mentor Smooth Round Plus Profile Saline, Style 1600). Several years later, the patient developed a left atrial myxoma, which was resected by cardiac surgery in May 2019 via minimally invasive right rib-spreading anterior minithoracotomy through the fourth intercostal space, with concurrent right breast prosthesis reimplantation ( Fig. 1 ).

In the summer of 2020, the patient developed sudden-onset right chest pain after vigorously embracing her son. Over time, she noted the contour of her right breast diminishing in size, worsening exertional dyspnea, and intermittent right-sided lower chest pain. On examination, asymmetry was noted on the anterior chest wall with a flattened right breast contour.

**Fig. 2:**
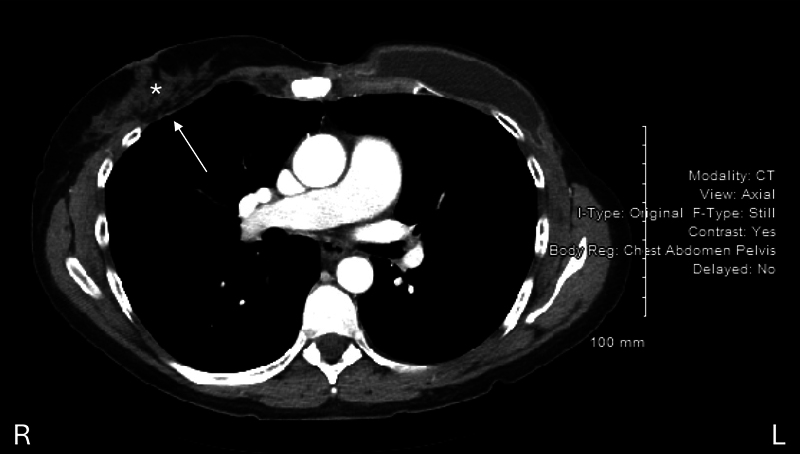
CT imaging demonstrating absence of the right prosthesis (*) with changes to the chest wall arrow).

**Fig. 3:**
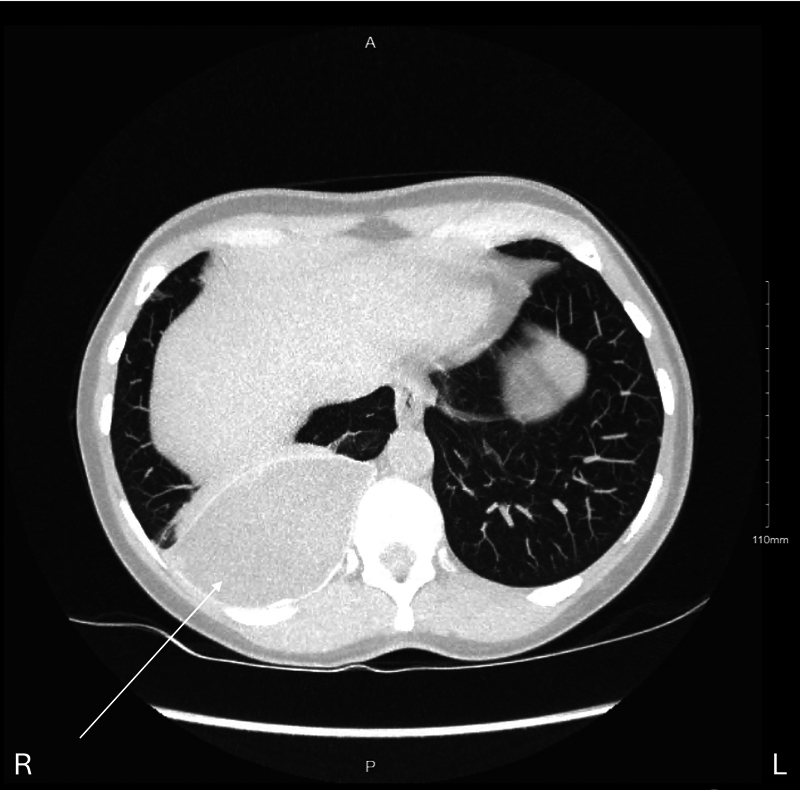
CT imaging demonstrating an intact breast prosthesis located in the right posterior basal pleural space (arrow).

In 2021, the patient was seen by a plastic surgeon to attempt removal of a suspected ruptured right breast implant. At the time of surgery, the implant was not found. It was noted that the patient had an anterior chest wall defect of approximately 5 cm with visible lung parenchyma. Subsequent CT imaging confirmed intrapleural migration of the right breast implant in the posterior right basal space, inferior to the right lower lobe ( Figs. 2 and 3 ), with the left-sided prosthesis remaining in situ. It is likely that the breast prosthesis migrated through the chest wall through the anterior chest wall defect after strong external mechanical pressure to the chest wall.

**Fig. 4:**
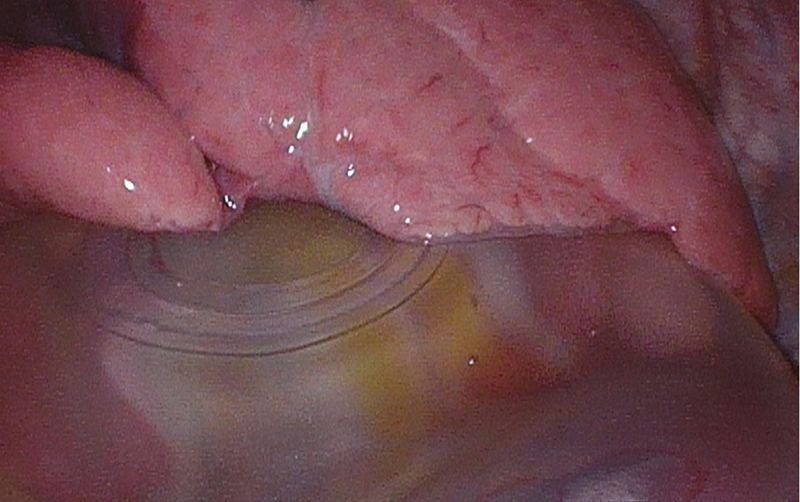
Thoracoscopic visualization of breast prosthesis adjacent to lung parenchyma.

The patient underwent right thoracoscopic exploration for retrieval of the migrated breast prosthesis. A right-sided mastectomy scar, a small right anterior thoracotomy incision, and two healed thoracoscopic port incisions were noted. An intact prosthesis was visualized thoracoscopically in the right posterior pleural space ( Fig. 4 ). The prosthesis was mobile and easily exteriorized through the utility incision. There were adhesions noted between the anterior chest wall and lung parenchyma at the location in the fourth intercostal space, where a slight weakness in the chest wall was palpable intraoperatively. Chest wall reconstruction was not felt to be necessary. At the time of this writing, the patient has not yet chosen to undergo breast prosthesis reimplantation.

## Discussion

Intrathoracic migration of breast prostheses is a rare but significant complication following chest wall, cardiac, or intrathoracic surgery in patients with a history of breast prosthesis implantation. To our knowledge, this is the first described case of a breast prosthesis migration after atrial myxoma resection with concurrent breast prosthesis reimplantation.

Implant migration is of concern to the clinician as it may have deleterious health consequences to the patient, including respiratory symptoms, pain, nausea, and decreased functional status, in addition to altered cosmetic appearance. In the reported literature, features concerning intrathoracic breast implant migration include sudden volume loss, new breast asymmetry, and new-onset shortness of breath. If implant migration is suspected based on history and exam findings, imaging is warranted in addition to the appropriate general investigations. While magnetic resonance imaging (MRI) is suitable for detecting implant migration as well as acute or chronic implant rupture, it may not be readily accessible or timely in all centers. A comprehensive initial evaluation to detect implant migration should include CT imaging. Optional preliminary chest radiography may be performed to detect suspicious intrathoracic densities or alternate symptom etiologies.

If migration occurs, the risk of simultaneous implant rupture has also been described, and lung herniation through the chest wall defect is possible. 2 4 9 11 Surgical strategies to mitigate the chance of breast prosthesis migration have been discussed previously, including avoiding disruption of the posterior implant capsule during chest wall or cardiac surgery. 4 8 14 If a chest wall defect is noted intra- or postoperatively, a mesh or tissue repair may be performed to repair this defect. 7 8 10 Surgeons should be aware of intact ipsilateral breast prostheses prior to any planned intrathoracic operation. If possible, the utility/access incision should be positioned remote from the implant. The incision should be kept at a minimal size, and muscle layers should be closed securely to minimize the chance of implant migration. 15

Although uncommon, intrathoracic migration of breast prostheses is a documented complication following cardiothoracic or breast surgery, which may occur in the initial postoperative setting or remotely from the surgery date. Anticipation of this potential complication will allow for appropriate pre- and intraoperative planning. Collaboration between surgical disciplines is vital to ensure risk mitigation and management of complications. Further research investigating how current surgical strategies impact the integrity of the chest wall and subsequent long-term complications in the patient with breast prostheses will be important to inform surgical planning and follow-up for this patient population.
